# Single intraoperative intravenous Co-Amoxiclav versus postoperative full oral course in prevention of postadenotonsillectomy morbidity: a randomised clinical trial

**DOI:** 10.1186/1472-6815-11-9

**Published:** 2011-09-30

**Authors:** Isaac M Macharia

**Affiliations:** 1 ENT department, Machakos General Hospital, Machakos, Kenya; 2 Department of otolaryngology, University of Nairobi, Nairobi, Kenya

## Abstract

**Background:**

Adenotonsillectomy results in postoperative morbidity which otolaryngologists attempt to reduce by use of antibiotics. The regimes used as quite varied with some opting for a full oral course postoperatively while others prefer prophylactic doses. This randomised clinical trial done in Kenyatta National Hospital, Kenya had the aim of comparing the efficacy of Co-Amoxiclav given as a single intravenous dose with a full oral course in the prevention of post adenotonsillectomy morbidity.

**Methods:**

126 patients below 12 years scheduled to undergo adenotonsillectomy were randomised into two groups. 63 were given a single intravenous dose of Enhancin [Co-Amoxiclav] at induction while the remaining half received a five days oral course of the same postoperatively. All received oral Pacimol [Paracetamol] in the postoperative period. Analysis was done and comparison made between the two groups with regards to pain, fever and diet tolerated in the postoperative period with a follow up period of seven days.

**Results:**

There was no statistical significant difference between the two groups with regards to postoperative pain, fever and diet tolerated. All had a P-value > 0.2. Postoperative pain was highest in the first postoperative day and reduced progressively to the lowest level on the 7^th ^postoperative day. As pain reduced, patients were able to tolerate a more solid diet with all but 6 tolerating their usual diet. 4 patients developed fever in the 1^st ^postoperative day which did not progress to the next day. One patient had fever on the 4^th ^and 7^th ^postoperative day and was admitted in the paediatrics' ward with a chest infection. All these patients with history of fever were in the group that was on oral postoperative Co-Amoxiclav.

**Conclusion:**

A single intraoperative dose of Co-Amoxiclav given intravenously at induction was found to be just as effective as a full oral course of the same given postoperatively in the prevention of post adenotonsillectomy morbidity. The prophylactic dose is favoured over the later as it is cheaper, ensures compliance and relieves off the need for refrigeration of the oral suspension as not all have access to refrigeration in low economy countries as ours.

**Trial registration:**

ClinicalTrials.gov: NCT01267942

## Background

Perioperative use of antibiotics has been shown to be effective in reducing some of the morbidities of adenotonsillectomy which include postoperative pain, fever and haemorrhage among others [1]. The odynophagia that results may limit food and fluid intake that could result in dehydration. Infection of the operation site has been thought to be a major contributor of the above for which otolaryngologists attempt to prevent by use of antibiotics.

Adenotonsillectomy is a clean contaminated operation for which prophylaxis is indicated. This aims at reducing microbial burden at the operation site as antibiotic is administered intravenously prior to contamination of previously sterile tissues [2,3]. This relieves the need to taking oral postoperative medication the compliance of which may be suboptimal post adenotonsillectomy due to odynophagia that results from traumatised pharyngeal tissues with possibility of resistance development.

Widespread and often inappropriate use of broad-spectrum antibiotics is responsible for development and spread of bacterial resistance [4]. Rational use of antibiotic is required if development of virulent microorganism is to be prevented. The current practice of antibiotic use with regards to adenotonsillectomy is not standardised as is evidenced by the varied regimes that have been used world wide [5]. Some otolaryngologists give postoperative oral antibiotics, some give few doses of intravenous antibiotics while others prefer no antibiotics. Many studies have been done with regards to perioperative antibiotic use with regards to adenotonsillectomy none of which was found to focus on prophylactic single preoperative intravenous antibiotic. This prompted us to do this study which compares prophylactic doses of Enhancin [Co-amoxiclav] with full postoperative oral courses of the same.

## Objective

To compare prophylactic doses of Co-Amoxiclav given at induction with postoperative full oral course of the same in their ability to reduce post adenotonsillectomy mordity.

## Methods

### Study design

Randomised clinical trial.

### Ethical approval

The protocol was reviewed and approved by Kenyatta National Hospital; Ethics and Research Committee. [P75/4/2008]

### Setting

Kenyatta National Hospital [Kenya], ENT department.

### Inclusion criteria

a. Patients below 12 years undergoing adenotonsillectomy.

b. Consenting parents or guardians of patients undergoing surgery.

### Exclusion criteria

a. Non-consenting parent or guardians of patients undergoing surgery.

b. Antibiotic use in the preceding week prior to admission.

c. Patients who develop complications that warranted change of antibiotic or required hospitalisation eg. Poor reversal from general anaesthesia.

d. Patients with co-morbid conditions like malnutrition, anaemia etc.

e. Known allergies to Penicillin or Co-Amoxiclav.

### Sample size determination

Sample size necessary to detect statistically significant difference between test and control groups in reduction of morbidity with Power of 80% and 5% significant level was calculated using the formula below by Cyrus R. Mehta [6].

$$2N=\frac{4{\left(Z_{\alpha }+Z_{\beta }\right)}^{2}\sigma^{2}}{\delta^{2}}$$

**2N **= Total number of patients in both arms of the study.

***Zα ***= 1.96 for type 1 error of 5%.

***Zβ ***= 0.842 for type 2 error 20%.

***σ ***= Variance from other studies.

***δ ***= Difference between test and control that is considered clinically significant.

From the calculations, a total number of 126 were required for the study; 63 in each arm.

### Trial design

The design was parallel with groups A and B. Group A patients were to receive intravenous Enhancin [Co-amoxiclav] while those in Group B were to receive postoperative Enhancin [Co-amoxiclav].

The principle investigator used Permuted-block randomisation with 2 blocks of equal size and an allocation ratio of 1:1. Randomisation was done within each group and allocation concealment achieved by sequentially-numbered opaque and sealed envelops.

The patients were recruited by the principle investigator and allocated their respective groups as per the content of the sequentially-numbered opaque sealed envelops.

## Statististical analysis

Data was analysed using the SPSS computer programme. The independent Student T test was used to compare data from the two groups with calculation of the t value for each of variables. This was subsequently used to calculate the Probability value, which would then determine significance of the observation made.

## Procedure

Research numbers were given to the patients consecutively from 1 to 126 once the study commenced. The principle researcher didn't participate in the surgeries. Both the surgeon and anaesthesiologist scheduled to perform the operation reviewed the patients preoperatively. The surgeon performing the surgeries was blinded but not the anaesthesiologist who gave the intravenous dose of Enhancin to the patients in Group A. Overnight admission was mandatory with starving of the patients six hours prior to surgery.

The patients were taken to the operating room the following day and Atropine was given half an hour before surgery to dry bronchial and oral secretions at a dose of 20 micrograms per kilogram [7].

Patients in group A received intravenous Enhancin [Co-Amoxiclav] at induction at a dose of 25 mg/kg and Pacimol [Paracetamol] suppositories 125 mg for patients under 5 years and 250 mg for those above 5 years [8]. Oral Pacimol [Paracetamol] was given to them in the postoperative period at a dose of 10 mg/kg [9].

Those in group B received Atropine and Pacimol [Paracetamol] at a similar timing and dose as those in group A. They however receive oral Enhancin [Co-Amoxiclav] for 5 days in the postoperative period at a dose of 25 mg/kg expressed as Amoxycillin [8]. No intraoperative Enhancin [Co-Amoxiclav] was given to patients in this group.

Tonsillectomy was done by blunt dissection while adenoidectomy by curettage. Diathermy or pressure packing achieved haemostasis. Documentation was done appropriately in forms that were made available in theatre.

The patients were discharged the day following surgery. Any complication in the general condition that arose was investigated and appropriate management given.

Patients, guardians or parents were advised to look out for haemorrhage, postoperative fever, severe odynophagia and vomiting in the postoperative period. They were advised to present themselves to hospital immediately for review in case there were changes in their general condition that gave concern. The principal researcher gave his mobile phone number to all those in the study and flash back services was used in case any information requirement arose.

### Outcomes

A blinded reviewer collected data for the outcomes listed below on the 1^st^, 4^th ^and 7^th ^postoperative days. He didn't know that the patients were split in two groups or the different medication given to either.

Comparison between the two groups was done with special emphasis on the outcomes below.

1. Postoperative pain was assessed using a visual analogue scale. The parents or guardians would be shown the scale and explained to on how it was used. They would then be asked to estimate the amount of pain they thought their patients were experiencing and indicate the same on a scale of 0 to 10 with 0 being 'no pain' and 10 being 'the worst imaginable pain'. This was assessed once on the 1^st^, 4^th ^and 7^th ^postoperative days.

2. Fever was defined by a temperature reading that was more than 37.2 degree on Celsius scale taken by thermometer in the axilla. A thermometer was placed in the axilla of the patient till it made an alert sound indicating that the maximum temperature had been attained.

Temperature was taken 4 hourly in the postoperative period till the patients were discharged and on presentation during the follow up period.

3. Return to normal diet was assessed by the duration of time taken for the patient to start taking his usual diet after surgery. A progressive assessment was done with regards on the diet consistency the patients were tolerating with the options of 'nil', 'liquid', 'semisolid' and 'usual'.

This was documented once on the days of review.

## Results

### Drop Out

There was a drop out of 10.6% as total of 141 patients were recruited into the study with 15 being excluded due to various reasons. The remaining 126 patients, 63 in each group were analysed.

The excluded had 5 patients in Group B who were given postoperative Amoxycillin instead of Enhancin [Co-Amoxiclav]. 2 patients in Group A were erroneously prescribed for postoperative oral Enhancin [Co-Amoxiclav] after they had already received the single intraoperative intravenous dose of the antibiotic. 8 patients failed to turn up for review, 4 in either group.

### Age

All the patients recruited into the study were below the age of twelve years with a mean of 4 years for both groups. The maximum age recruited for Group A was 7 years and 9 years for Group B. The minimum age was 1.5 years for either groups.

### Sex

More males were recruited into the study than females in both groups. 36 of the 63 in Group A were male forming 57% while they were 74% in Group B as 47 of the 63 were of the said gender.

### Indication For Surgery

In order to standardise the study, only patients undergoing both adenoid and tonsillar surgery were recruited. Thus the indications for surgery fell in two groups. One group had obstructive symptoms due to adenoid and tonsillar hypertrophy while the other group had chronic recurrent tonsillitis and adenoid hypertrophy. Group A had 50 of the 63 with adenotonsillar hypertrophy while Group B had 53 of the 63 with this indication. This translates to 79% and 84% in Group A and Group B respectively.

### Analysis of pain

The visual analogue scale was used to evaluate pain the patients were experiencing postoperatively at day 1, 4 and 7.

Patients in both groups reported a high score in the first postoperative day with reduction of the same with all reporting lowest levels at day 7.

Comparison between the two groups was made using the Students T test [independent sample test] at day 1, 4 and 7.

In all the days analysed, thresholds required to reject the null hypothesis were not reached. Thus, with regards to pain, there was no demonstrable difference between a single dose of intravenous Enhancin [Co-Amoxiclav] given at induction and a full oral postoperative course of the same.

### Diet

The diet that the patients could take was assessed at day 1, 4 and 7. Generally it was noted that as the days passed after the surgery, the patients could tolerate more solid feeds as is expected.

There were no statistical significant differences between the two groups.

The Students T test [independent sample test] was used to compare the groups with regards to feeds on subsequent days. The two groups were not found to have any statistical difference with regard to feeds on all the days that were analysed. Thus there was no demonstrable difference between a single intravenous dose of Co-Amoxiclav given at induction and a full oral postoperative course of the same with regards to resumption to usual diet after operation.

### Temperature

No patient had fever on the day of the operation in both groups. On the first postoperative day 4 patients all in group B had fever. None in group A had fever.

On the 4^th ^postoperative day, only 1 patient in group B had fever. This patient was not among those who had fever on the 1^st ^postoperative day. No patient in group A had fever.

On the 7^th ^postoperative day, the same patient who had presented with fever on the 4^th ^postoperative day had fever. This patient was admitted in the paediatric ward the following day with a chest infection. He had taken Enhancin [Co-Amoxiclav] and Pacimol [Paracetamol] for five days as had been instructed.

Analysis of the two groups with regards to temperature was made with the Students T test [independent sample test]. There was no statistical significant difference between the two groups. It can thus be deduced that there is no difference between a single intravenous dose of Enhancin [Co-Amoxiclav] given at induction and a full oral postoperative course of the same in prevention of postoperative infection.

For all of the outcomes analysed, none was found to have statistically significant difference (Table 1).

**Table 1:** Table representing outcome.

Outcome	Pain			Diet			Temperature		
**Day**	**1**	**4**	**7**	**1**	**4**	**7**	**1**	**4**	**7**

Tvalue	-0.09	1.489	-0.60	-1.08	0.779	0.832	-2.05	-1.00	-1.00

Df	124	124	124	124	124	124	124	124	124

Pvalue	>0.2	>0.2	>0.2	>0.2	>0.2	>0.2	>0.2	>0.2	>0.2

Significance	Nil	Nil	Ni	Nil	Nil	Nil	Nil	Nil	Nil

## Discussion

Fever in the postoperative period could be due to infection or as a response to tissue injury. Both conditions give rise to pyrogenic cytokines that cause production of Prostaglandin E 2 with resetting of the hypothalamic thermal setpoint. Interleukin 2 has been identified as an important intracellular messenger in the development of fever whether due to trauma or infection [10].

Fever that develops during the first two days postoperatively is usually regarded to be due to the body's response to trauma while that which develops later is likely to have an infective pathophysiology. 4 patients developed fever in the first postoperative day, which by the second day had resolved. All belonged to group B and were on oral Enhancin [Co-Amoxiclav] and Pacimol [Paracetamol]. All had low-grade fever as none had temperature more than 38 degrees Celsius. They formed 3.1% of all the patients in the study compared to 54% in V.T Anand's study of postoperative fever in paediatrics after tonsillectomy [11]. In the later study, temperature was monitored 2 hourly after surgery in the first 24 hours while it was monitored 4 hourly in this current study with documentation starting the day after surgery. This could mean that some patients might have had developed fever but not captured as monitoring in the first 24 hours was not done comprehensively. It was also not considered whether Dexamethasone was or not given during the surgery as is the practice of some anesthesiologists. This could suppress the inflammatory response in the first 24 hours therefore reducing those with fever in the immediate postoperative period.

The effect of Pacimol [Paracetamol] should also be taken into account due to its antipyretic activity. It was not considered whether the antipyretic was given before or after the temperatures were taken.

Only 1 patient had fever at day 4 and 7. He belonged to group B and was on oral Enhancin [Co-Amoxiclav] and Pacimol [Paracetamol] and didn't have fever at day 1. He was admitted in the paediatric ward and treated for pneumonia with intravenous drugs and discharged 5 days later.

Analysis of Enhancin [Co-Amoxiclav] given as a single dose at induction and the same given orally postoperatively show no difference in their ability to prevent postoperative infection, which presents clinically as fever.

Pain in the postoperative period result from trauma to the surrounding tissues during surgery, diathermy or ligation especially if the constrictors are involved. Infection gives rise to inflammation, which causes increase in pain being experienced by a patient. Pain in the first two postoperative days is due to trauma to the surrounding tissues while an increase in pain at a later day may be a pointer to infection. Pain may restrict feeds and fluids intake that may result in dehydration. Compliance to oral medication in the postoperative period could be suboptimal due to odynophagia with selection of resistant microorganisms. In the study, pain was noted to be highest in the first postoperative day and least on the 7^th^. It is expected that in the absence of infection, pain should decrease with progressive healing of the tissues which allows the patients to tolerate more solid feeds as the discomfort in the oropharynx reduces.

The patient who developed a chest infection on the 4^th ^postoperative day was on soft feeds on the said and 7^th ^day. He was not able to take his usual diet on the 7^th ^day. He didn't have infection in the tonsillar region and the inability to take his usual diet could have been a misinterpretation of anorexia, which is an attendant of respiratory tract infection.

Analysis of the two groups with regards to postoperative pain and diet showed no statistical significance. This means that a single dose of Enhancin [Co-Amoxicav] given at induction is just as effective as a full oral course of the same given postoperative period with regards to pain and diet, which in effect reflects on healing.

Colveary M.P et al. did a study on the postoperative effect of Amoxycillin/Clavulinic acid in post tonsillectomy patients with regards to postoperative pain and diet tolerated. They compared a group of patient given the antibiotic orally postoperatively with patient not given. Antibiotic use was found to be useful in reducing morbidity postoperatively as judged by the amount of analgesics used (P = 0.379), time to resume normal diet (P = 0.0072) and visual analogue scale (P = 0.0006) [1]. They didn't consider postoperative fever in their study. Fennesby B.G et al found level 1 evidence that antibiotics reduce the incidence of postoperative fever, oropharyngeal pain and return to normal activity post tonsillectomy [12]. The authors had done literature review through Pubmed in an attempt to identify the level of evidence for or against the use of prophylaxis for various ear, nose and throat surgeries. These, in addition to other studies confirms that antibiotics reduce morbidity post tonsillectomy. Thus adenotonsillectomy patients not given antibiotics are expected to present with a higher incidence of postoperative fever, pain and take longer to tolerate their normal diet. Our study has shown that both a prophylactic dose and full postoperative dose of antibiotic have equal efficacy in the prevention of postadenotonsillectomy morbidity. Had a third arm been added to the study in which patients were not given antibiotics, more pain, higher incidence of fever and delayed return to normal diet is to be expected. These studies differ with ours as we focus on prophylaxis with regards to adenotonsillectomy. Colveary'study though titled: 'Antibiotic prophylaxis post-tonsillectomy is it of benefit' doesn't pass as prophylaxis as the antibiotic was given orally postoperatively. The principles of prophylaxis require the antibiotic to be given intravenously at induction as a stat dose so as to have adequate concentrations in the tissues before incision [13]. Studies that were found to use single doses of antibiotic didn't pass as prophylactic either as the antibiotic was either given orally preoperatively or postoperatively. No study was found that focused on single intravenous preoperative antibiotic in the reduction of postoperative morbidity as this is the requirement for qualification as prophylaxis.

It is due to this that we think that our study sheds more light with regards to prophylaxis which the study has proven to be just as effective as postoperative full oral course.

## Shortcomings of the Study

Blinding of the parents/patients was not done. This could have had an effect on the subjective outcomes like pain and diet tolerated. The objective outcome of temperature was not affected by the lack of blinded stated above.

## Conclusions

From the study's results and their analysis, a single intraoperative dose of Enhancin [Co-Amoxiclav] given at induction is just as effective as full oral postoperative course of the same in prevention of post adenotonsillectomy morbidity.

The fore mentioned medication is cheaper as it is given once and compliance is assured. Emergence of resistant and virulent microorganisms is prevented by this prophylactic medication, as compliance to oral medication in patients post adenotonsillectomy is not assured due to odynophagia.

Oral Enhancin [Co-Amoxiclav] requires refrigeration once reconstituted so as to ensure potency. As refrigeration might not be available in some of the homes of the patients, a single dose at induction circumvents the predicament.

A prophylactic single dose of Enhancin [Co-Amoxiclav] given at induction is thus recommended.

## Competing interests

The authors declare that they have no competing interests.

## Authors' contributions

M.D developed the study from the inception of the idea to full execution, analysis and interpretation of the results while I.M participated in the design and interpretation of the results. Both authors read and approved the final draft.

## Pre-publication history

The pre-publication history for this paper can be accessed here:

http://www.biomedcentral.com/1472-6815/11/9/prepub
